# Severe Tick Infestation and *Haemogregarina* spp. Infection in a Captive African Rock Python (*Python sebae*): A Clinical and Parasitological Case from Nigeria

**DOI:** 10.1002/vms3.70571

**Published:** 2026-06-30

**Authors:** Foluke Adedayo Akande, Adrian A. Diaz‑Sanchez, Adeniyi Olugbenga Egbetade, Iskiil Oladehinde Oyenekan, Adeniyi Adekunle Adebiyi, Iyanu David Oyemade, Timothy Bamgbose, Angélique Foucault‑Simonin, Alejandro Cabezas‑Cruz

**Affiliations:** ^1^ College of Veterinary Medicine Federal University of Agriculture Abeokuta Ogun State Nigeria; ^2^ Department of Biology University of Saskatchewan Saskatoon Saskatchewan Canada; ^3^ Department of Biological Sciences Microbiology Unit Kings University Odeomu Osun Nigeria; ^4^ Anses, INRAE École Nationale Vétérinaire d'Alfort UMR BIPAR Laboratoire de Santé Animale Maisons‑Alfort France

**Keywords:** *Amblyomma latum*, ectoparasites, *Haemogregarina* spp. infections, *Python sebae*, reptile health, tick infestation

## Abstract

Ectoparasitic infestations in reptiles—primarily ticks and mites—can significantly compromise their health and fitness, particularly in captivity conditions. This brief report describes a severe tick infestation and concurrent *Haemogregarina* spp. infection in an African rock python (*Python sebae*) at a private zoo in Abeokuta, Nigeria. Initial clinical evaluation revealed heavy infestations of *Amblyomma latum* ticks, pronounced facial swelling, incomplete shedding, anorexia, lethargy, and the presence of intraerythrocytic *Haemogregarina*‐like gamonts on stained blood smears. Haematological analysis further showed mild haemoconcentration and elevated leukocyte differentials suggestive of an inflammatory response, potentially caused by a parasitic infection. The treatment protocol consisted of mechanical removal of ticks, oral administration of Ivermectin, and improved husbandry practices to prevent ectoparasite re‐infestation. Post‐treatment, the python showed a notable clinical improvement, evidenced by resumed feeding and normal shedding within five days. Microscopic examination of blood smears post‐treatment confirmed the absence of *Haemogregarina*‐like gamonts, indicating the effectiveness of the therapeutic approach. Overall, the present case report highlights the importance of parasitic surveillance, rapid intervention, and adherence to rigorous husbandry standards to ensure the health and welfare of reptiles in captivity.

## Graphical abstract

Heavy *Amblyomma  latum* infestation transmitted haemoparasites to a captive African rock python, causing anorexia, facial swelling and dysecdysis. Manual tick removal, povidone‐iodine cleansing and a single oral dose of ivermectin cleared both ecto‐ and haemoparasites. Rapid recovery and parasite‐free blood smears highlight the need for routine ectoparasite control and haemoparasite surveillance in captive reptiles.

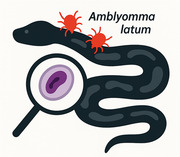

## 1 Introduction

The African rock python (*Python sebae*), one of the largest non‐venomous constrictor snake species in the world, is native to sub‐Saharan Africa (O'Shea 2007). Although not currently endangered, this species faces ongoing threats in Nigeria from habitat loss, hunting, and human‐wildlife conflict in moist forests and savannahs (Akani et al. 1999). Reptiles, found globally, represent a highly diverse vertebrate class, yet relatively little is known about the diversity and systematics of their parasites compared with those of mammals and birds (Al‐Quraishy et al. 2021). Furthermore, the diversity of haemoparasites in reptiles—at both the genera and species levels—exceeds that found in mammals and birds (Abdel‐Baki et al. 2014; Úngari et al. 2022), likely due to factors such as limited mobility, specialised habitats, and the evolutionary longevity of reptiles (Ball 1967).

The cosmopolitan phylum Apicomplexa Levine 1970 includes parasites of both medical and veterinary importance, many of which are transmitted by arthropod vectors and infect a wide array of hosts ranging from invertebrates to humans (Levine 1970). Within this phylum, the family Haemogregarinidae Neveu‐Lemaire, 1901, comprises coccidial blood parasites commonly referred to as haemogregarines (Mohammed and Mansour 1959). This family includes three genera—*Cyrilia* Lainson, 1981*, Desseria* Siddall, 1995, and *Haemogregarina* Danilewsky, 1885 (Barta 1989; Siddall 1995). Particularly, *Haemogregarina* species are the most common sporozoan intracellular haemoparasites in reptiles, infecting both aquatic and terrestrial hosts, and are mainly transmitted by leeches, mosquitoes, and ticks (Telford 2009).

In snakes, transmission of haemogregarines often results from the ingestion of the definitive invertebrate host or, more frequently, an infected intermediate host such as insectivorous lizards and frogs (Tomé et al. 2012; Desser et al. 1995). The identification of haemogregarine species is challenging, relying primarily on the morphology and morphometrics of the intraerythrocytic gamonts observed in peripheral blood smears of the vertebrate host (Hull and Camin 1960; Telford 2009). Although haemogregarine infections in reptiles are usually considered apathogenic, their impact on the vertebrate host's health remains poorly understood and can range from asymptomatic to severe clinical disease (Brown et al. 2006). This brief report describes an atypical case presentation of haemogregarine infection in a captive African rock python (*Python sebae*) from Nigeria, emphasising the importance of effective health management in captive reptiles.

## 2 Materials and Methods

### 2.1 Case Presentation

On 14 February 2020, a private zoo in Abeokuta, Nigeria, requested veterinary consultation for an African rock python that had been anorexic for one week, exhibiting unusual facial swelling along with marked reduction in activity. The python was individually housed on wood chips and pebbles within a 1.5 m^3^ enclosure constructed of concrete‐and‐glass with ventilation openings and was fed a weekly diet of mice and chicks. Upon visual examination, the patient was found to be heavily infested with ticks, primarily around the head region, and showed signs of incomplete shedding, as reported by handlers (Figure 1). The sex and age of the patient were not specified; facility records indicated that it had been acquired from external vendors and kept at the zoo for nearly 2 years. Other reptiles and animals were kept separately in the same facility.

**FIGURE 1:**
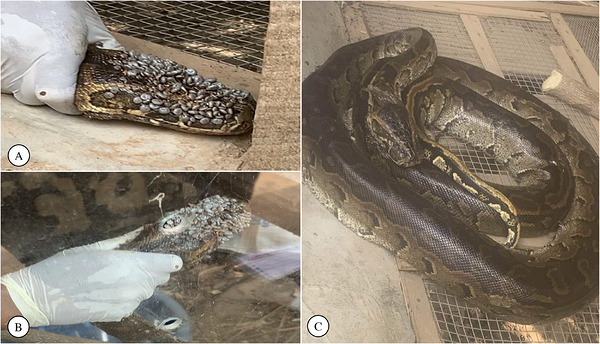
African rock python (*Python sebae*) before (A–B) and after (C) treatment. (A–B) Before treatment, the python exhibited pronounced facial swelling, heavy tick infestation, and incomplete shedding. (C) Post‐treatment, the python showed resolved swelling, absence of ticks, and normal shedding, reflecting a successful recovery.

### 2.2 Clinical and Parasitological Examination

#### 2.2.1 Patient Immobilisation

The python was subjected to physical and clinical examinations, weighed, and measured. The patient was immobilised following the protocol described by Girling and Raiti (2004), using a combination of ketamine HCl (250 mg) and diazepam (5 mg), administered at 25 mg and 0.5 mg per kg body weight, respectively.

#### 2.2.2 Tick Collection

The collected ticks were washed, sorted, and preserved in 70% ethanol for further taxonomic identification, following the methodology described by Walker (2003).

#### 2.2.3 Blood Sample Collection

A blood sample was collected via cardiocentesis using a sterile 23‐gauge hypodermic needle attached to a 3 mL syringe. After collection, three to five thin blood smears were prepared, while the remaining blood sample was stored in EDTA tubes, refrigerated at 4°C, and analysed within 24 h for haematological analyses.

#### 2.2.4 Microscopy

The blood smears were air‐dried, fixed in absolute methanol for 3 min, and stained with 10% Giemsa Methylene Blue Eosin Merck stain solution (Merck, USA), diluted in distilled water (pH 7.0) for 50 min (Eisen and Schall 2000). All blood smears were examined for parasite detection at a final magnification of 1000× under a Leica DM4000 B compound light microscope, equipped with a Leica DFC310 FX 1.4‐megapixel digital colour camera and LAS V3.8 software (Leica Microsystems, Germany). Identification of the tick was done using a Leica EZ4 dissecting stereomicroscope (Leica Microsystems, Germany) according to the standard taxonomic keys described by Estrada‐Peña et al. (2004).

#### 2.2.5 Haematological Analysis

PCV, total WBC count, and differential counts (heterophils, eosinophils, lymphocytes, and monocytes) were evaluated. PCV was determined by microhaematocrit centrifugation at 15,000 × g for 5 min (Sorvall Legend Micro 21R Microcentrifuge, Thermo Scientific, USA), with results read via a DAMON/IEC haematocrit card reader. Total WBC count was manually performed using Neubauer counting chambers (Neubauer Improved, Germany) at 400× magnification under a Leica DM4000 B microscope (Leica Microsystems, Germany). Differential counts were conducted on Giemsa‐stained blood smears at 1000× magnification, with results converted to absolute counts (occurrence per 100 cells × total leucocyte count). Reference intervals from Jegede et al. (2017) for African rock pythons (*Python sebae*) were used for clinical assessment.

#### 2.2.6 Treatment

The python was treated with 10% Povidone Iodine (Sawke, Kattle Care) and relocated to a tick‐free enclosure. Post‐anaesthesia, oral ivermectin (0.2 mg) was administered due to its safety in pythons and effectiveness against ectoparasitic infestations.

## 3 Results

### 3.1 Tick Infestations and Identification

All tick specimens were identified morphologically as *Amblyomma latum* (Figure 2). The python measurement and tick distribution are described in Table 1.

**FIGURE 2:**
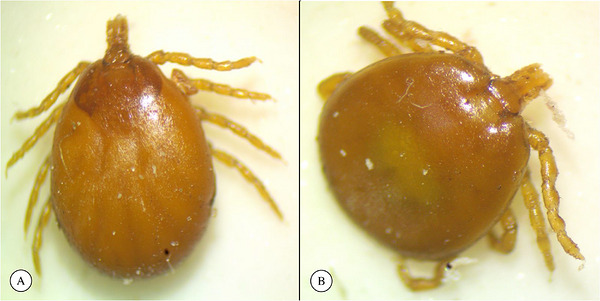
Adult stages of *A. latum*. (A) Female. (B) Male. Close‐up images of the ticks collected from the African rock python (*Python sebae*), illustrating key morphological features—such as scutal shape, mouthpart length, and the absence of eyes—used to confirm the species as *A. latum* according to standard taxonomic keys.

**TABLE 1:** Showing the distribution of ticks all over the python as well as the length and weight.

Parameter	Measurement/Count
Python length	∼9 feet
Python weight	10 kg
Total ticks collected	754
Ticks from head region	382
Ticks from other body areas	372
Tick distribution (head region)	
Larvae	2
Nymphs	195
Adults	185
Males	99
Females	86
Tick distribution (other body areas)	
Larvae	5
Nymphs	81
Adults	286
Males	94
Females	192

### 3.2 Microscopic Findings

Microscopic examination of the thin blood smears revealed the presence of intraerythrocytic gamonts with distinctive disposition, shape, morphological, and nuclear arrangement, which are concordant with haemogregarine infection in the python's red blood cells (Figure 3).

**FIGURE 3:**
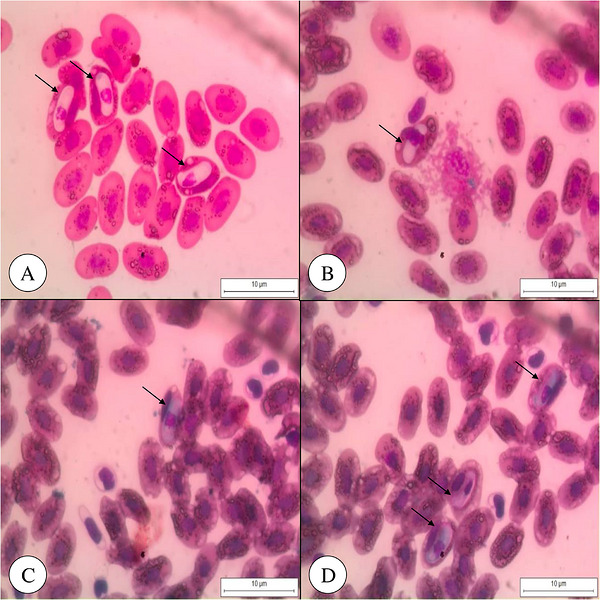
(A–D) Representative Giemsa‐stained thin blood smears from an African rock python (*Python sebae*), with mature intraerythrocytic haemogregarine gamonts (arrows) within nucleated red blood cells, observed under 1000× magnification. The haemogregarines appear as dark, elongated bodies with basophilic cytoplasm and small, deeply stained nuclear regions. In some erythrocytes, the parasites displace or partially overlap the nucleus and may be present singly or in pairs. Haemogregarine infections in snakes are diagnosed through microscopic examination of thin blood smears, revealing these characteristic intraerythrocytic gamonts. Each scale bar represents 10 µm.

### 3.3 Haematological Findings

The haematological analysis revealed a PCV of 37%, a WBC of 27 × 10⁹ cells/L, and differential counts of 18.10 × 10^9^ cells/L for heterophils, 7.83 × 10^9^ cells/L for lymphocytes, 0.54 × 10^9^ cells/L for monocytes, and 0.54 × 10^9^ cells/L for eosinophils (Table 2).

**TABLE 2:** Haematological findings in the python.

Parameter	Value	Reference interval (RI)
PCV (%)	37	16–26
WBC count (×10⁹ cells/L)	27	7.10–29.40
Heterophils (×10⁹ cells/L)	18.10	4.26–21.76
Lymphocytes (×10⁹ cells/L)	7.83	2.50–8.20
Monocytes (×10⁹ cells/L)	0.54	0.00–0.44
Eosinophils (×10⁹ cells/L)	0.54	0.00–0.59

### 3.4 Treatment Effect

Following initial treatment, the patient showed marked clinical improvement within 72 hours, consistent with recovery times reported in the literature (Mader 2006). Remarkably, the python completed a full shed within 5 days after tick removal, indicating a swift return to normal ecdysis and physiological function.

## 4 Discussion

As shown in Figure 1, characteristic haemogregarine gamonts were found within the cytoplasm of erythrocytes; these oval, darkly stained body inclusions are consistent with intraerythrocytic gamonts of the genus *Haemogregarina*. In ophidian hosts, cytological identification of haemogregarine species often relies on the detection of their parasitic stages inside the erythrocytes (Hull and Camin 1960). The large size and prominent nuclei of reptilian erythrocytes can influence the haemoparasites morphology, challenging their cytological identification compared to those in mammals and birds (Ursula et al. 2014).

The assessed haematological parameters showed a PCV value higher than the reference range of 16–26%, indicating mild haemoconcentration or dehydration, consistent with the initial clinical symptom of anorexia. The WBC count was near the upper limit, suggesting subclinical infection or a stress response. Differential counts revealed heterophils and lymphocytes near their upper limits, while monocytes were slightly elevated, and eosinophils remained within the normal range. These haematological findings—monocytosis combined with borderline eosinophilia—suggest the presence of a mild inflammatory response, potentially caused by both haemogregarine infection and heavy *A. latum* infestation (Stahl 2006).

*A. latum*—commonly known as the snake tick—is a hard tick species with broad distribution throughout sub‐Saharan Africa (Guglielmone and Robbins 2018). This Afrotropical tick frequently parasitises reptiles and can act as a biological vector of haemoprotozoan pathogens, including haemogregarines and *Hepatozoon* spp. (Kenny et al. 2004). In snakes, all active developmental stages of *A. latum* may feed on one host, with adults typically attached under the dorsal and lateral scales across the body, where the head is often the preferred attachment site (Nowak 2010). In captivity, reptiles face a higher risk of recurrent tick infestations because limited space and restricted movement allow ticks to complete their life cycle and re‐infest the same host (Castro, Pietzsch, and Pantchev 2019). These ectoparasite infestations can cause dysecdysis and poor nutritional status, often accompanied by wounds and scars (Natusch et al. 2018).

In reptiles, ectoparasitic infestations can lead to both immediate and long‐term health issues, with negative impacts on overall host health and fitness through direct and indirect mechanisms still poorly understood (Argaez et al. 2020). This brief report highlights the value of specialised veterinary care for effective management of ectoparasitic and haemoparasitic infections in captive exotic reptiles. The authors further recommend to sanitary authorities that such information should be considered into future protocols for reptile captivity and conservation programmes.

## 5 Conclusion

This report documents the first case of *Hepatozoon pythonis* in an African rock python and reveals the impact of severe tick infestation. The successful management of the case reinforces the importance of early diagnosis, proper veterinary intervention, and improved husbandry practices in reptile health care.

## Author Contributions

**Foluke Adedayo Akande**: conceptualisation, project administration, supervision, writing – original draft, writing – review and editing. **Adrian A. Díaz‐Sánchez**: investigation, writing – original draft, writing – review and editing. **Adeniyi Olugbenga Egbetade**: investigation, writing – review and editing. **Iskiil Oladehinde Oyenekan**: investigation, writing – review and editing. **Timothy Bamgbose**: formal analysis, writing – review and editing. **Angélique Foucault‐Simonin**: writing – review and editing. **Alejandro Cabezas‐Cruz**: conceptualisation, writing – original draft, writing – review and editing. **Adeniyi Adekunle Adebiyi**: investigation, writing – review and editing. **Iyanu David Oyemade**: investigation, writing – review and editing.

## Consent

The authors have nothing to report.

## Conflicts of Interest

The authors declare no conflicts of interest.

## Data Availability

The data generated during this study are available upon reasonable request.

## References

[vms370571-bib-0001] Abdel‐Baki, A. A. , S. Al‐Quraishy , and J. Y. Zhang . 2014. “Redescription of *Haemogregarina garnhami* (Apicomplexa: Adeleorina) From the Blood of *Psammophis schokari* (Serpentes: Colubridae) as *Hepatozoon Garnhami* n. comb. Based on Molecular, Morphometric and Morphologic Characters.” Acta Parasitologica 59, no. 2: 294–300. 10.2478/s11686-014-0241-3. 24827101

[vms370571-bib-0002] Akani, G. C. , I. F. Barieenee , D. Capizzi , and L. Luiselli . 1999. “Snake Communities of Moist Rainforest and Derived Savanna Sites of Nigeria: Biodiversity Patterns and Conservation Priorities.” Biodiversity and Conservation 8: 629–642. 10.1023/a:1008849702810.

[vms370571-bib-0003] Al‐Quraishy, S. , F. Abdel‐Ghaffar , M. A. Dkhil , and R. Abdel‐Gaber . 2021. “Haemogregarines and Criteria for Identification.” Animals 11, no. 1: 170. 10.3390/ani11010170. 33445814 PMC7828249

[vms370571-bib-0004] Argaez, V. , I. Solano‐Zavaleta , and J. J. Zúñiga‐Vega . 2020. “Do Ectoparasites Affect Survival of Three Species of Lizards of the Genus *Sceloporus*?” Zoology 138: 125723. 10.1016/j.zool.2019.125723. 31756648

[vms370571-bib-0005] Ball, G. H. 1967. “Some Blood Sporozoans From East African Reptiles.” The Journal of Protozoology 14, no. 2: 198–210. 10.1111/j.1550-7408.1967.tb01983.x. 4962564

[vms370571-bib-0006] Barta, J. R. 1989. “Phylogenetic Analysis of the Class Sporozoea (Phylum Apicomplexa Levine, 1970): Evidence for the Independent Evolution of Heteroxenous Life Cycles.” Journal of Parasitology 75, no. 2: 195–206. 10.2307/3282766. 2494316

[vms370571-bib-0007] Brown, G. P. , C. M. Shilton , and R. Shine . 2006. “Do Parasites Matter? Assessing the Fitness Consequences of Haemogregarine Infection in Snakes.” Canadian Journal of Zoology 84, no. 5: 668–676. 10.1139/z06-044.

[vms370571-bib-0008] Castro, P. D. J. , M. Pietzsch , and N. Pantchev . 2019. “Ectoparasites in Captive Reptiles.” The Veterinary Nurse 10: 33–41.

[vms370571-bib-0009] Desser, S. S. , H. Hong , and D. S. Martin . 1995. “The Life History, Ultrastructure, and Experimental Transmission of *Hepatozoon Catesbianae* n. comb., an Apicomplexan Parasite of the Bullfrog, *Rana catesbeiana*, and the Mosquito, *Culex territans*, in Algonquin Park, Ontario.” Journal of Parasitology 81, no. 1: 212–222. 10.2307/3283922. (April). 7707196

[vms370571-bib-0010] Eisen, R. J. , and J. J. Schall . 2000. “Life History of a Malaria Parasite (*Plasmodium mexicanum*): Independent Traits and Basis for Variation.” Proceedings of the Royal Society B: Biological Sciences 267, no. 1445: 793–799. 10.1098/rspb.2000.1073. PMC1690602 10819149

[vms370571-bib-0011] Estrada‐Peña, A. , A. Bouattour , J. L. Camicas , and A. R. Walker . 2004. Ticks of Domestic Animals in the Mediterranean region: A Guide to Identification of Species. University of Zaragoza.

[vms370571-bib-0012] Girling, S. , and P. Raiti . eds. 2004. BSAVA Manual of Reptiles. British Small Animal Veterinary Association, 3rd ed. British Small Animal Veterinary Association (BSAVA).

[vms370571-bib-0013] Guglielmone, A. A. , and R. G. Robbins . 2018. Hard Ticks (Acari: Ixodida: Ixodidae) Parasitizing Humans: A Global Overview. Springer International Publishing.

[vms370571-bib-0014] Hull, R. W. , and J. H. Camin . 1960. “Haemogregarines in Snakes: The Incidence and Identity of the Erythrocytic Stages.” Journal of Parasitology 46, no. 4 (August): 515–523. 10.2307/3275151. 14403945

[vms370571-bib-0015] Jegede, H. O. , T. O. Omobowale , B. S. Okediran , and A. A. Adegboye . 2017. “Hematological and Plasma Chemistry Values for the African Rock Python (*Python sebae*).” International Journal of Veterinary Science and Medicine 5, no. 2: 181–186. 10.1016/j.ijvsm.2017.10.001. 30255069 PMC6137848

[vms370571-bib-0016] Kenny, M. J. , S. E. Shaw , P. D. Hillyard , and A. B. Forbes . 2004. “Ectoparasite and Haemoparasite Risks Associated With Imported Exotic Reptiles.” Veterinary Record 154, no. 14: 434–435. 10.1136/vr.154.14.434. 15119896

[vms370571-bib-0017] Levine, N. D. 1970. “Taxonomy of the Sporozoa.” Journal of Parasitology 56: 208–209.

[vms370571-bib-0018] Mohammed, A. H. H. , and N. S. Mansour . 1959. “The Haemogregarine Complex (an analytical systematic review).” Bulletin of the Faculty of Science, Cairo University 35: 39–51.

[vms370571-bib-0019] Mader, D. R. 2006. Reptile Medicine and Surgery. Saunders Elsevier.

[vms370571-bib-0020] Natusch, D. J. D. , J. A. Lyons , S. Dubey , and R. Shine . 2018. “Ticks on Snakes: The Ecological Correlates of Ectoparasite Infection in Free‐ranging Snakes in Tropical Australia.” Austral Ecology 43, no. 5: 534–546. 10.1111/aec.12590.

[vms370571-bib-0021] Nowak, M. 2010. “The International Trade in Reptiles (Reptilia)—The Cause of the Transfer of Exotic Ticks (Acari: Ixodida) to Poland.” Veterinary Parasitology 169, no. 3–4: 373–381. 10.1016/j.vetpar.2010.01.006. 20153933

[vms370571-bib-0022] O'Shea, M. 2007. Boas and Pythons of the World. Princeton University Press.

[vms370571-bib-0023] Siddall, M. E. 1995. “Phylogeny of Adeleid Blood Parasites With a Partial Systematic Revision of the Haemogregarine Complex.” Journal of Eukaryotic Microbiology 42, no. 2: 116–125. 10.1111/j.1550-7408.1995.tb01551.x. 7757052

[vms370571-bib-0024] Stahl, S. J. 2006. “Reptile Hematology and Serum Chemistry.” Veterinary Clinics of North America: Exotic Animal Practice 9: 321–332.16759950

[vms370571-bib-0025] Telford, S. R. 2009. Hemoparasites of the Reptilia: Color Atlas and Text. CRC Press, Taylor & Francis Group. 10.1201/9781420080414.

[vms370571-bib-0026] Tomé, B. , J. P. M. C. Maia , and D. J. Harris . 2012. “ *Hepatozoon* Infection Prevalence in Four Snake Genera: Influence of Diet, Prey Parasitemia Levels, or Parasite Type?” Journal of Parasitology 98, no. 5: 913–917. 10.1645/GE-3111.1. 22551400

[vms370571-bib-0027] Úngari, L. P. , E. C. Netherlands , A. L. Q. Santos , et al. 2022. “Diversity of Haemogregarine Parasites Infecting Brazilian Snakes From the Midwest and Southeast Regions With a Description of Two New Species of *Hepatozoon* (Apicomplexa: Adeleorina: Hepatozoidae).” Parasitology International 89: 102587. 10.1016/j.parint.2022.102587. 35452797

[vms370571-bib-0028] Ursula, H. , R. Korbel , F. Mutschmann , and M. Rinder . 2014. “Blood Parasites in Reptiles Imported to Germany.” Parasitology Research 113: 4587–4599. 10.1007/s00436-014-4149-5. 25324132

[vms370571-bib-0029] Walker, A. R. 2003. “Ticks of Domestic Animals in Africa: A Guide to Identification of Species.” Bioscience Reports.

